# Clinical Predictors of Nondiabetic Kidney Disease in Patients with Diabetes: A Single-Center Study

**DOI:** 10.1155/2021/9999621

**Published:** 2021-07-12

**Authors:** Francesco Fontana, Rossella Perrone, Francesco Giaroni, Gaetano Alfano, Silvia Giovanella, Giulia Ligabue, Riccardo Magistroni, Gianni Cappelli

**Affiliations:** ^1^Struttura Complessa di Nefrologia e Dialisi, Azienda Ospedaliero-Universitaria di Modena, Modena, Italy; ^2^Surgical Medical and Dental Department of Morphological Sciences, University of Modena and Reggio Emilia, Modena, Italy

## Abstract

**Background:**

Although diabetic kidney disease (DKD) could affect up to one-third of patients with diabetes mellitus (DM), these patients can develop kidney diseases different from DKD, or these conditions can superimpose on DKD. Several potential predictors of nondiabetic kidney disease (NDKD) have been proposed, but there are no definitive indications available for kidney biopsy in diabetic patients.

**Methods:**

We designed a single-center, cross-sectional, and retrospective cohort study to identify clinical and laboratory factors associated with a diagnosis of NDKD after native kidney biopsy in diabetic patients and to investigate differences in time to end-stage kidney disease (ESKD) in patients with a diagnosis of DKD and NDKD.

**Results:**

Of 142 patients included in our analysis, 89 (62.68%) had a histopathological diagnosis of NDKD or mixed NDKD + DKD. Patients in the NDKD group had significantly lower HbA1C, lower prevalence of diabetic retinopathy (DR), and less severe proteinuria, and there was a lower proportion of patients with nephrotic syndrome; the DKD group had significantly lower proportion of patients with hematological conditions. In the multivariate binary logistic regression, only absence of DR and presence of a hematological condition significantly predicted NDKD after adjustment for age and sex. Time to ESKD was significantly higher in patients with NDKD or mixed forms than in those with DKD.

**Conclusions:**

After a careful selection, more than half of kidney biopsies performed in diabetic patients can identify NDKD (alone or with concomitant DKD). Absence of DR and coexistence of a hematological condition (especially MGUS) were strong predictors of NDKD in our cohort.

## 1. Introduction

The global burden of diabetes mellitus (DM) has substantially increased in the last decades, mostly due to a rise in the prevalence of type 2 DM secondary to obesity and the metabolic syndrome [1]. Approximately 25 to 30 percent of diabetic patients will develop diabetic kidney disease (DKD), and DM currently represents the leading cause of chronic kidney disease (CKD) and end-stage kidney disease (ESKD) worldwide [1, 2], with expected consequences in patients' morbidity and mortality. Hyperglycemia-induced microvascular changes in the kidney are responsible for the development of diabetic nephropathy, or diabetic kidney disease (DKD), a condition with distinctive histopathological features (i.e., glomerular and tubular basement membrane thickening, mesangial matrix expansion with nodule formation, hyaline material accumulation in the glomerular capillary loops, and arteriolar hyalinosis) [1, 3]. Although novel therapeutic approaches are emerging or under study [4], treatment of diabetic nephropathy presently largely relies on nonspecific interventions, with therapeutic goals consisting in maintaining a tight glycemic control, using renin-angiotensin-aldosterone inhibitors to reduce glomerular hyperfiltration and proteinuria, and ensuring adequate control of blood pressure and other cardiovascular risk factors. Despite being defined as a histopathological entity, the diagnosis of DKD is most often based only on clinical and laboratory parameters, and a kidney biopsy is generally not required for confirmation, even if this would be the only way to determine if kidney dysfunction is a direct consequence of the diabetic environment [1]. The cardinal factors considered as associated with DKD in diabetic patients are the progression from microalbuminuria to overt proteinuria over time and a (relatively slow) decrease in glomerular filtration rate (GFR), after a phase of hyperfiltration, generally with an enlarged normal-sized kidney on ultrasound [3].

Diabetic patients can develop kidney diseases different from DKD, or these conditions can superimpose on DKD; since clinical presentation is generally nonspecific (kidney dysfunction and proteinuria), clinicians are less prone to perform diagnostic kidney biopsy in these patients and could erroneously provide a presumptive diagnosis of DKD, limiting the access to specific treatments. The indication to kidney biopsy in a patient with DM and kidney dysfunction and/or proteinuria represents a clinical conundrum, and several studies have attempted to identify clinical and laboratory factors associated with a histopathological diagnosis of nondiabetic kidney disease (NDKD) in order to restrict biopsy to patients with a high probability of being candidates to different therapeutic interventions. Clinically accepted predictors of NDKD in diabetic patients are the absence of DR, sudden onset of nephrotic syndrome or nephrotic-range proteinuria, short duration of diabetes, microscopic hematuria (especially with dysmorphic erythrocytes), and low glycated hemoglobin levels [5]. However, the generalizability of the aforementioned findings might be low in selected populations (for instance, in a recent meta-analysis of 48 studies, more than a half reported data on patients of Asian descent [6]), and there are no definitive indications available for kidney biopsy, often leaving the decision to single-center policies.

The performance of kidney biopsy in diabetic patients has also been proved to provide prognostic information; indeed, patients with DKD have been reported to have shorter renal survival compared to patients with NDKD [7–9]. Although data on patient survival are less uniformly reported, a recent study identified a diagnosis of DKD as a risk factor for mortality [7].

The aim of our study was to investigate the clinical and laboratory factors associated with a diagnosis of NDKD in a cohort of patients with DM who underwent kidney biopsy on clinical indication. We also investigated differences in renal and patient survival among patients with DKD or NDND.

## 2. Materials and Methods

We designed a single-center, retrospective, cross-sectional, and cohort study to identify clinical and laboratory factors associated with a histopathological diagnosis of NDKD after native kidney biopsy in diabetic patients (including under the term NDKD also patients with mixed forms, NDKD + DKD) and to investigate differences in time to ESKD in patients with a diagnosis of DKD and NDKD after kidney biopsy. We enrolled all adult patients with a diagnosis of type I or type II DM who underwent diagnostic native kidney biopsy on clinical indication at the Nephrology and Dialysis Unit, University Hospital of Modena, Modena, Italy, from January 1, 2010, to April 30, 2020. The study was approved by the local ethical committee (“Comitato Etico dell'Area Vasta Emilia Nord” (protocol no. 252/2020)).

Data were collected from clinical charts and histopathological reports; patients with incomplete clinical or laboratory data or with inadequate samples in kidney biopsy were excluded from the analysis. The following clinical variables at kidney biopsy were considered: age, sex, body mass index (BMI), blood pressure, presence of nephrotic syndrome, acute kidney injury, need for dialysis, type of diabetes, presence of DR, diabetes duration, presence of hematological conditions, and history of cardiovascular diseases (previous myocardial infarction or stroke). Laboratory data at biopsy included the following: serum creatinine and estimated GFR (eGFR) according to the CKD-EPI equation, 24 h proteinuria or proteinuria/creatininuria on a single-void urine sample, serum albumin, presence of microhematuria, serum cholesterol levels, serum complement levels (C3/C4), antinuclear antibody (ANA) and antineutrophil cytoplasm antibody (ANCA) positivity, antiphospholipase A2 receptor (PLA2R) positivity, viral serologies for hepatitis B virus (HBV), hepatitis C virus (HCV), and human immunodeficiency virus (HIV), and glycated hemoglobin (HbA1C). Nephrotic syndrome was defined as the combination of proteinuria of more than 3.5 g per day, hypoalbuminemia (less than 3.5 g/dl), and edema. Acute kidney injury was defined according to the KDIGO criteria [10]. We defined “hematological condition” as the presence of monoclonal gammopathy of undetermined significance (MGUS), monoclonal B-cell lymphocytosis (MBL), or multiple myeloma (MM).

Renal biopsy was performed on clinician discretion, and indications included the following (alone or in combination): nephrotic syndrome (especially if of sudden onset and in patients without DR), subnephrotic proteinuria in patients with a DM duration of less than 5 years, rapidly progressive kidney dysfunction with or without urinary abnormalities, and acute kidney injury without convincing clinical explanation. Histological examination, performed by one of two expert nephropathologists, was conducted on light microscopy for Bouin medium-fixed paraffin-embedded tissue with standard staining and immunofluorescence analysis for fresh frozen tissue with standard antisera; electron microscopy analysis was performed in a minority of cases on clinician indication and if dedicated material was available. Histological features of DKD were diffuse mesangial sclerosis with or without nodule formation and glomerular basement membrane thickening; supportive features were tubular basement membrane thickening (in nonatrophic tubules), presence of glomeruli (fibrin caps and capsular drop), and arteriolar hyalinosis.

Statistical analysis was performed using GraphPad Prism (version 6.01) and SPSS (version 23) software. Continuous data were reported as median and interquartile range or mean and standard deviation as appropriate and discrete data as absolute number and relative percentage; data were compared with Student's *t*-test, Kruskal–Wallis test, and chi-square test as appropriate. Univariate binary logistic regression was used to identify independent factors predictive of NDKD; a multivariate logistic regression model adjusting for age and sex was then developed including all variables with *p* < 0.1. Differences in time to ESKD (defined as the need of renal replacement treatment, RRT) and death among the DKD group and the NDKD and mixed DKD + NDKD group were estimated with the Kaplan–Meier method with log-rank test.

## 3. Results

**Figure 1:**
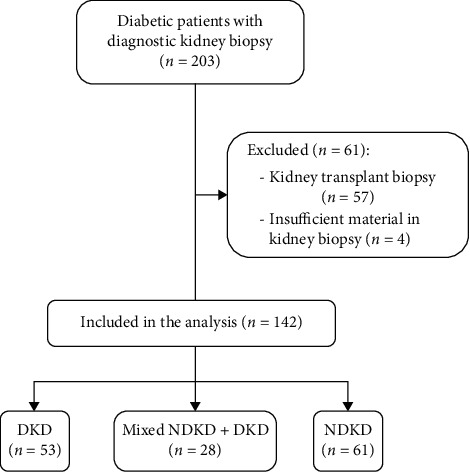
Study design and patients' selection.

We screened 203 diabetic patients who underwent diagnostic kidney biopsy at our center; after exclusion of biopsies performed in kidney-transplanted patients and of cases with insufficient material for a diagnosis, 142 patients were included in our analysis. Of them, 53 patients (37.32%) had a diagnosis of DKD in kidney biopsy, 28 (19.72%) of NDKD with mixed DKD, and 61 (42.96%) of isolated NDKD (see Figure 1). Clinical and laboratory data at the time of kidney biopsy are summarized in Table 1. The average age of patients was 62.65 ± 12.35 years, and most were males (72.5%) and of Caucasian descent (88.03%); average duration of DM was 11.9 ± 9.7 years. Diabetic patients who received kidney biopsy presented with significant kidney dysfunction, with an average eGFR of 36.2 ± 27.02 ml/min, and urinary abnormalities (median proteinuria/creatininuria: 3.895 g/g, IQR: 1.195–6.925, and microhematuria in 62.68% of patients). In the DKD group, there was a lower proportion of Caucasian patients (79.2%), while patients in the NDKD group had significantly lower systolic blood pressure, shorter duration of diabetes (median: 6 years; IQR: 3–11), lower HbA1C levels (43.5 mmol/mol; IQR: 38.2–53.7), and lower prevalence of DR (13.1%). The NDKD group showed less severe proteinuria, with lower proteinuria/creatininuria (median: 2 g/g; IQR: 0.6–4.3), higher serum albumin levels (3.5 g/dl, IQR: 3.0–4.0), and lower proportion of patients with nephrotic syndrome (27.9%). Of note, there were no differences between the groups in the proportion of patients presenting with microhematuria. Interestingly, a significantly lower proportion of patients with DKD compared with other groups carried a diagnosis of a hematological condition. Conversely, we encountered no differences among the groups in the proportion of patients who had a positive serology for chronic viral infections or autoimmune diseases nor in the levels of serum complement fractions C3 and C4.

**Figure 2:**
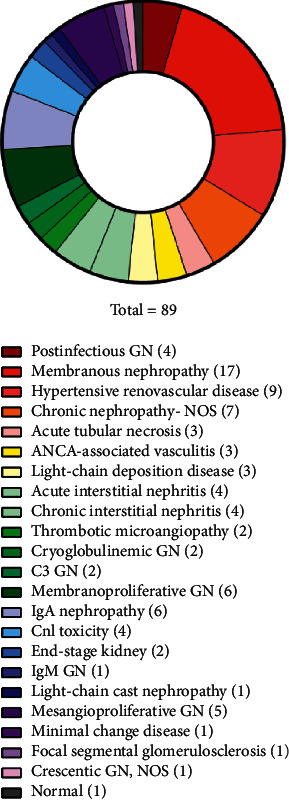
Kidney biopsy histological diagnosis in patients with NDKD or mixed NDKD + DKD. NOS: not otherwise specified; GN: glomerulonephritis.

Eighty-nine diabetic patients received a main histological diagnosis different from DKD (in 28 of them, there was coexistence of NDKD and DKD). The most frequent diagnosis in the NDKD and NDKD + DKD groups was membranous nephropathy (19.1%), hypertensive renovascular disease (8.99%), chronic nephropathy—not otherwise specified (7.87%), membranoproliferative glomerulonephritis (not further specified) (6.74%), and IgA nephropathy (6.74%). The complete list of diagnosis in the NDKD and NDKD + DKD groups is depicted in Figure 2. Of the 89 patients who received a main diagnosis of NDKD, 46 revealed histological lesions that altered treatment decision, prompting specific (immunosuppressive) treatment.

Twenty patients had a hematological condition, mostly benign; the majority of them (85%) had a diagnosis of MGUS, 2 had MM, and 1 had MBL; 2 patients with MGUS also had cryoglobulinemia (of unspecified type). Histological diagnosis obtained from kidney biopsy of the 20 patients with hematological conditions and NDKD is reported in Table 2.

To identify independent predictors of NDKD in kidney biopsy, we tested several factors in a preliminary univariate analysis. The following variables were included in the final multivariate model: diabetes duration, nephrotic syndrome, DR, hematological condition, and HbA1C. After adjustment for age and sex, only the absence of DR and the presence of a hematological condition maintained significance in predicting the specified outcome (see Table 3).

**Figure 3:**
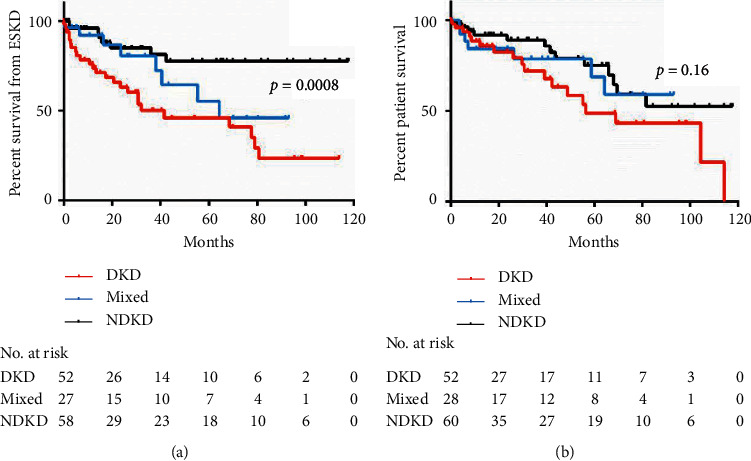
Kaplan–Meier curves for time to end-stage kidney disease (ESKD) (a) and time to death (b).

With respect to long-term kidney outcome of our cohort, roughly one-third of patients (*n* = 45) developed ESKD during follow-up. We encountered a higher proportion of patients needing RRT in the DKD group (*n* = 26, 49%) with respect to the NDKD (*n* = 10, 16%) and the mixed groups (*n* = 9, 32%); four patients (1 in the DKD, 1 in the mixed, and 2 in the DKD groups) were on dialysis at the time of kidney biopsy. Time to ESKD was significantly shorter in the DKD group, as shown by the Kaplan–Meier curves in Figure 3(a) (log-rank test, *p*=0.0008). We did not identify difference in patient survival comparing the two groups (see Figure 3(b)).

## 4. Discussion

In our cohort of 142 diabetic patients who required kidney biopsy, NDKD was identified in 62.68% of patients (with 61 patients having isolated NDKD and 28 NDKD and coexistent DKD). We found that the absence of DR and the presence of a hematological condition were the strongest predictors of NDKD as a main histopathological diagnosis.

Nephropathy represents a frequent microvascular complication of DM, with up to one-third of patients being reported to develop DKD in the course of their life. Nevertheless, these patients can develop other types of kidney disease, which require a different diagnostic and therapeutic approach, and the frequency of these conditions in diabetic patients is reasonably expected to grow with the steady increase in the prevalence of DM worldwide.

With the present study, we showed that, in a carefully selected population of diabetic patients, over a half received a histological diagnosis of kidney pathology different from DKD, allowing for a more precise diagnostic and prognostic classification and, in a relevant proportion of cases, opening the opportunity for specific treatments. Regarding this aspect, our findings are in line with those from a recent study from Spain including more than 800 diabetic patients (mostly Caucasians), where NDKD and mixed NDKD + DKD were diagnosed, respectively, in 49.6% and 10.8% [7]; similarly, previous studies from the U.S. [11] and China [12] identified alternative diagnosis to DKD in more than 50% of cases. These data confirm that even if precise indications for kidney biopsy in diabetic patients are lacking, clinical judgment based on generally accepted parameters represents a fair discriminator; nevertheless, still a high proportion of biopsies yields a diagnosis of DKD and potentially could have been avoided, at least from the patients' perspective.

Regarding the histopathological findings in the NDKD and mixed NDKD + DKD groups, in our cohort, the most frequent diagnosis was membranous nephropathy (almost 20% of cases in these groups). This appears somewhat conflictive with the data from the aforementioned larger Spanish court [7], where hypertensive nephrosclerosis was the dominant finding alternative to DKD. A possible explanation for this difference is that since most diabetic patients are also hypertensive and considering that renal histological features of the two conditions can easily overlap (for instance, arteriolosclerosis or arteriolar hyalinosis), we were very cautious in proposing nephrosclerosis as a main diagnosis if DKD features, even minimal, were present. Supporting our findings, membranous nephropathy was also the most frequent glomerular disease described in a previous Italian study [13]. Interestingly, many studies reported IgA nephropathy as the main diagnosis alternative to superimposed DKD [8, 9, 14, 15]. We consider that, among other factors, differences in the ethnic background (i.e., proportion of patients of Asian descent) could account for this lack of homogeneity. Of note, compared with the data from Sharma et al. [11], extrapolated from a large U.S. diabetic population, we had a consistently lower number of diagnoses of acute tubular injury, possibly reflecting also a substantial difference in kidney biopsy indications among different countries.

Diabetes duration and levels of HbA1C have been reported as important predictive factors for DKD in previous studies [9]; nevertheless, we were not able to confirm this finding in our cohort. Indeed, while our patients in the NDKD group showed a shorter duration of diabetes and lower HbA1C levels, the significance of these elements to predict the histological diagnosis was not maintained in the logistic regression after adjustment for other covariates. We hypothesize that the lack of significance of these factors, which should be logically associated with a higher “diabetes burden,” could be ascribed to the relatively small size of our cohort. It must also be considered that self-reported duration of diabetes could be imprecise and that patients could be diabetic for years before being diagnosed as such. In sum, we presently tend to consider these elements as of limited clinical utility, at least in our diabetic population.

Similarly, the degree of proteinuria and the presence of nephrotic syndrome, although significantly different among the groups we examined (with patients with DKD presenting with higher proteinuria and more frequent nephrotic syndrome), did not predict NDKD in the multivariate analysis. Regarding this aspect, similar results were reported from Sharma et al. [11]. This finding underlines the absence of specificity in the presentation of different kidney diseases, an extremely common concept in clinical nephrology, and the main reason justifying the use of kidney biopsy to obtain a precise diagnosis. Certainly, these considerations strongly apply to diabetic patients also with kidney dysfunction and proteinuria. Due to the lack of specific data (before kidney biopsy) in a considerable number of patients, we were not able to assess the impact of the sudden onset of nephrotic syndrome in the final histopathological diagnosis. Indeed, this factor could have been of interest since it is commonly considered as suggestive for NDKD, as opposed to the typical progression of DKD from the stage of microalbuminuria through overt proteinuria and eventually to nephrotic syndrome.

Diabetic patients with NDKD have been reported to show a slightly higher prevalence of microhematuria, especially with dysmorphic erythrocytes, and this factor has been claimed as a potential predictor of NDKD [16]. We did not identify differences between our groups in the proportion of patients with microhematuria, and we presently consider it as a rather unspecific finding in patients with DM and kidney dysfunction and/or proteinuria. Moreover, a recent study on 261 patients with biopsy-proven DKD demonstrated the presence of hematuria in almost half of them [17]; interestingly, hematuria was associated with more severe histological damage and predicted a worse prognosis in this cohort.

Serological tests, such as markers of chronic viral infections, autoimmunity, and serum complement levels, are often used in clinical practice to refine the indication to kidney biopsy; this is considered especially useful in patients with metabolic conditions such as diabetes and hypertension, which can per se lead to kidney dysfunction and urinary abnormalities. Nevertheless, in our study, groups did not differ for positive autoimmune (ANA, ANCA, and anti-PLA2R) or viral serologies (HBV, HCV, and HIV). We acknowledge that the relatively low number of patients under study could complicate the identification of small differences between groups; however, similar results were obtained in a previous study involving a larger cohort [11]. Even if, from the clinical standpoint, the positivity of these elements could logically trigger a lower “threshold” for kidney biopsy in diabetic patients, this behavior is not presently corroborated by solid scientific data.

Conversely, our data suggest that the absence of DR in a diabetic patient with kidney dysfunction and urinary abnormalities substantially strengthens the indication to kidney biopsy. In our cohort of diabetic patients, the absence of DR significantly predicted NDKD. Indeed, it has been previously reported that DR has a high sensitivity for predicting DKD [18], and in a recent meta-analysis of 48 studies, this factor was one of the most consistently associated with DKD among the studies considered [6].

We believe that the most relevant finding of our study is the association between the presence of a hematological condition and NDKD in kidney biopsy, which has not been clearly reported before. The occurrence of kidney diseases associated with hematological conditions, especially monoclonal gammopathies, in the absence of symptomatic MM or chronic lymphocytic leukemia is increasingly recognized [19]. The term monoclonal gammopathy of renal significance (MGRS) has been created to identify these cases, and the classification of MGRS is in continuous evolution, requiring the deployment of different histopathological techniques (electron microscopy analysis and immunofluorescence after pronase digestion), often not widely available. Even if it is already accepted among nephrologists that benign hematological conditions could cause significant kidney pathology, our study provides further evidence for considering MGUS (and similar disorders), in the appropriate clinical setting, as possibly associated with kidney diseases other than DKD in diabetic patients. Certainly, diabetic patients with coexisting hematological conditions and kidney dysfunction and/or proteinuria could especially benefit from kidney biopsy for diagnostic discrimination. We acknowledge that a minority of our patients with hematological condition and NDKD had a histological diagnosis that directly demonstrated kidney involvement secondary to the condition itself. Nonetheless, a number of diagnoses of membranoproliferative glomerulonephritis, chronic nephropathy—not otherwise specified—and membranous glomerulonephritis were not further analyzed due to the lack of electron microscopy examination and impossibility to perform immunofluorescence after pronase digestion of tissue to identify masked monotypical deposits [20]. Indeed, this could have led to a possible diagnostic misclassification, inappropriately labeling a process as not secondary to the hematological condition. Whether the presence of MGUS could be associated with renal diseases different from MRGS, possibly representing an epiphenomenon of an autoimmune disorder is presently not known.

We also highlighted a faster progression of CKD in patients with DKD, compared with those with NDKD or mixed forms. This has been previously reported [7] and is likely related to the fact that a significant proportion of patients with NDKD could be proposed specific treatments, which can slow disease progression. On the contrary, it is also relevant to note that, considering these data, a diagnosis of DKD in kidney biopsy implies a worse kidney prognosis compared to NDKD. For this reason, patients with DKD should be offered close monitoring and intensive treatment to slower the progression of kidney damage (i.e., strict glycemic control, judicious use of renin-angiotensin-aldosterone blockers, aggressive blood pressure control, and, whenever possible, new drugs that demonstrated to improve renal prognosis, such as SGLT-2 inhibitors). We were not able to detect a decrease in patient survival in the DKD group; indeed, our cohort was small and with a relatively short follow-up.

Our study has several limitations. Importantly, an inherent selection bias is evident since patients who were presumed to have a high probability of DKD were excluded a priori, not being offered kidney biopsy. Nevertheless, our data represent an insight into the common practice of clinical nephrologists and could apply to the population of diabetic patients who are considered for kidney biopsy. We also acknowledge that our study was monocentric, with a retrospective design and including a relatively small number of patients, factors limiting the generalizability of our findings.

## 5. Conclusions

In summary, our study confirms that, if carefully selected, more than a half of kidney biopsies performed in diabetic patients can identify NDKD (alone or with concomitant DKD). The strongest predictors of NDKD in kidney biopsy in our cohort of diabetic patients were the absence of DR and the coexistence of a hematological condition (especially MGUS), and clinicians should maintain a low threshold for kidney biopsy if these characteristics are met. Patients with DKD progressed more rapidly to ESKD than patients with NDKD or mixed forms; aggressive supportive therapy to slower the progression of CKD should be warranted if DKD is identified in kidney biopsy.

## Figures and Tables

**Table 1:** Clinical and laboratory characteristics of patients with DKD, NDKD, and mixed NDKD + DKD.

Variables	All patients	DKD	Mixed NDKD + DKD	NDKD	*p* value
No. of patients (%)	142	53 (37.32)	28 (19.72)	61 (42.96)	–
Age (yr)	62.65 ± 12.35	59.9 ± 12.2	63.9 ± 12.6	64.5 ± 12.1	0.12
Males (%)	103 (72.5)	39 (73.6)	21 (75.0)	43 (70.5)	0.89
Race, Caucasians (%)	125 (88.03)	42 (79.2)	26 (92.8)	57 (93.4)	0.045
Type I DM (%)	5 (3.52)	3 (5.6)	1 (3.6)	1 (1.6)	0.5
BMI	28.7 ± 5.7	28.1 ± 5.1	30.0 ± 7.5	28.7 ± 5.4	0.36
Systolic blood pressure (mmHg)	138.7 ± 15.5	142.2 ± 16.2	138.5 ± 17.1	135.9 ± 13.7	0.09
Diastolic blood pressure (mmHg)	77.7 ± 9.6	77.8 ± 9.7	79.2 ± 8.5	76.9 ± 10.0	0.59
Diabetes duration (yr)	11.9 ± 9.7	14 (7–20)	12 (6.2–18.7)	6 (3–11)	<0.001
Diabetic retinopathy (%)	49 (34.51)	32 (60.4)	9 (32.1)	8 (13.1)	0.001
Ischemic heart disease/stroke (%)	52 (36.62)	22 (41.5)	11 (39.3)	19 (31.1)	0.53
Smokers (%)	25 (17.73)	10 (18.9)	5 (17.9)	10 (16.4)	0.95
HbA1C (mmol/mol)	51.0 ± 14.55	49 (42–66)	50.5 (42.2–56.5)	43.5 (38.2–53.7)	0.04
Proteinuria/creatininuria	3.895 (1.195–6.925)	5 (3.6–8.5)	5.2 (0.9–9.6)	2 (0.6–4.3)	<0.001
eGFR (ml/min)	36.2 ± 27.02	34.4 ± 33.2	31.9 ± 16.7	39.7 ± 33.2	0.37
Microhematuria (%)	89 (62.68)	34 (64.2)	21 (75.0)	34 (55.7)	0.21
s-albumin (g/dl)	3.329 ± 0.76	3.2 (2.8–3.7)	3.24 (2.7–3.8)	3.5 (3.0–4.0)	0.07
Hypercholesterolemia (%)	82 (57.75)	32 (60.4)	18 (64.3)	32 (52.5)	0.57
s-hemoglobin (g/dl)	11.09 ± 2.0	10.8 ± 1.6	11.0 ± 1.7	11.4 ± 2.4	0.36
Nephrotic syndrome (%)	59 (41.55)	28 (52.8)	14 (50.0)	17 (27.9)	0.02
Acute kidney injury (%)	49 (34.51)	16 (30.2)	9 (32.1)	24 (39.3)	0.6
s-C3 (mg/dl)	113.4 ± 17.1	114.6 ± 19.7	115.3 ± 33.3	111.5 ± 26.7	0.77
s-C4 (mg/dl)	26.78 ± 10.3	29.4 ± 10.2	25.3 ± 10.2	25.1 ± 10.2	0.06
ANA or ANCA or anti-PLA2R+ (%)	22 (15.49)	5 (9.4)	3 (10.7)	14 (23)	0.1
HBsAg or HCV or HIV+ (%)	19 (13.38)	4 (7.5)	4 (14.3)	11 (18)	0.26
Hematological conditions (MGUS, MM, and MBL) (%)	21 (14.79)	2 (3.8)	6 (21.4)	13 (21.3)	0.02

**Table 2:** Kidney biopsy histological diagnosis of patients with hematological conditions.

Histopathological diagnosis	Number of cases
Membranous glomerulonephritis	3
Hypertensive renovascular disease	3
Light-chain deposition disease	3
Diabetic kidney disease	2
Chronic nephropathy—not otherwise specified	2
Membranoproliferative glomerulonephritis	2
Postinfectious glomerulonephritis	1
Chronic interstitial nephritis	1
IgA nephropathy	1
IgM nephropathy	1
Cast nephropathy	1

**Table 3:** Multivariate logistic regression model for the prediction of NDKD as a main diagnosis at kidney biopsy.

Variables	OR (95% CI)	*p* value
Age	1.017 (0.982–1.053)	0.356
Sex	0.667 (0.250–1.781)	0.419
Diabetes duration	0.969 (0.928–1.012)	0.153
Nephrotic syndrome	0.673 (0.282–1.604)	0.371
Diabetic retinopathy	0.183 (0.076–0.446	0.0001
Hematological condition	8.903 (1.047–75.725)	0.045

## Data Availability

The data used to support the findings of this study are stored in the digital archive of the “Azienda Ospedaliero-Universitaria di Modena” and are available from the corresponding author upon request.
